# Gender Biases in the Accuracy of Facial Judgments: Facial Attractiveness and Perceived Socioeconomic Status

**DOI:** 10.3389/fpsyg.2022.884888

**Published:** 2022-05-31

**Authors:** Yue Qi, Jia Ying

**Affiliations:** ^1^Department of Psychology, Renmin University of China, Beijing, China; ^2^Graduate School of Education, University of Pennsylvania, Philadelphia, PA, United States

**Keywords:** facial attractiveness, gender bias, impression, socioeconomic status, facial judgment

## Abstract

Many studies demonstrate that people form their first impression of a stranger based on facial appearance, and these impressions influence their subsequent decisions and behaviors. However, much less research has examined the factors that moderate the accuracy of first impressions based on a photo of face. The present study included three experiments to explore gender differences in the accuracy of impressions based on faces. The results showed that people judge facial attractiveness more accurately for female faces than for male faces while giving more accurate wealth judgments for male faces than for female faces. Interestingly, although we did not find a significant correlation between confidence ratings and the accuracy of wealth rating, we recognized a significant moderate correlation between confidence ratings and the accuracy of attractiveness ratings when female participants rated male faces. To our knowledge, the present study is the first to reveal gender biases in the accuracy of impression judgments based on facial appearance. These findings imply a significant influence of traditional gender roles on accurate facial judgments.

## Introduction

When interacting with a stranger, people may form their first impression based on limited available information (e.g., facial appearance), and these judgments can subsequently and indirectly influence social decision making (Qi et al., 2018, 2021; Li et al., 2021). Many studies demonstrate that facial attractiveness has an impact on various social decisions, such as friendship and mating choices (Thornhill and Gangestad, 1999), monetary decision-making (Pandey and Zayas, 2021), and hiring (Luxen and Van De Vijver, 2006). People judge facial attractiveness based on common aesthetic or affective attributes of different genders (Rhodes, 2006). According to the owner hypothesis, facial attractiveness is a stable characteristic of those with faces (Chen et al., 1997; Little and Perrett, 2002). Researchers have explored some facial features that affect facial attractiveness judgments, such as averageness (Komori et al., 2009), symmetry (Baudouin and Tiberghien, 2004), sexual dimorphism (Perrett et al., 1998; Russell, 2003), and vitality (Zheng and Zhou, 2021). The observer hypothesis argues the importance of the beholder on facial attractiveness perception and emphasizes the characteristics of the observer, such as the observer’s age (Little et al., 2010), personality (Welling et al., 2009), and sociocultural factors (Little et al., 2002). Attractiveness can be a sign of health, and highly attractive faces can induce positive and pleasant emotional experiences (Rhodes, 2006; Zhang et al., 2021), which are rewarding to individuals (Aharon et al., 2001). Previous studies have found that the reward value of facial attractiveness can be influenced by the gender of the perceiver (Cloutier et al., 2008; Levy et al., 2008).

Facial gender is another impact factor in attractiveness processing. Mitrovic et al. (2018) found that both males and females looked longer at female faces, especially attractive female faces. This is in accordance with the “female beauty captures the mind” hypothesis (Maner et al., 2003). From an evolutionary perspective, males and females will emphasize the different characteristics of potential mates. Males pay more attention to characteristics related to reproductive potential, such as physical attractiveness, while females pay more attention to characteristics that signal resource acquisition, such as status and dominance (Buunk et al., 2002). Furthermore, attractive female faces capture more behavioral attention (Slater et al., 1998; Maner et al., 2003), bring more rewards (Collins and Missing, 2003; Colwell, 2007; Wang et al., 2015), and cause more brain activation in neural mechanisms (Zhang et al., 2012; Ru et al., 2017). In other words, attractive female faces capture more attention and are more visible than attractive male faces.

To date, many studies have discussed the accuracy of facial judgments (e.g., Todorov et al., 2015; Walker and Vetter, 2016). However, much less research has examined the factors that moderate the accuracy of first impressions when viewing a photo of a face (Alaei and Rule, 2016). Previous studies have investigated self-other agreement on traits in face-to-face contexts and found that extroversion and openness can be accurately judged (e.g., Borkenau et al., 2009; Back et al., 2010; Moritz and Roberts, 2018). However, neuroticism is the least accurately judged trait in online contexts (Gosling et al., 2007; Back et al., 2010). These findings can be explained by the trait visibility effect (Funder and Dobroth, 1987); that is, the more relevant and frequent the behaviors the trait elicits, the more accurate the judgments that are made will be (Watson et al., 2000), because perceivers can acquire more valid cues to judge the trait.

Moreover, previous studies have revealed that the longer people know each other, the more accurately they rate each other’s traits. Compared with strangers who observed behaviors for only a few minutes, acquaintances predicted behavior better and were more consistent with their reports of observed behavior (Biesanz et al., 2007). For example, married couples have higher self-other agreements on most affectivities and personalities than friendship dyads or dating couples do (Watson et al., 2000). Increased acquaintanceship is accompanied by more trait-relevant messages; thus, perceivers can make more accurate judgments of the target (Funder, 1995; Funder et al., 1995). Considering that facial attractiveness carries additional significance for women (Luxen and Van De Vijver, 2006), people may be more accustomed to evaluating women’s attractiveness in everyday life. Thus, we expected gender bias in the accuracy of attractiveness judgment from faces.

The present study was designed to explore the influence of gender factors on the accuracy of people’s judgments of facial attractiveness. In the review of Tsankova and Tair (2021), the accuracy of first impressions refers to “the correspondence between the subjective perception of the interaction partners and some more objective criterion (e.g., Funder and West, 1993; Brauer and Proyer, 2020).” Thus, previous research commonly uses the term “accuracy” to illustrate the agreement between actual cooperative behaviors or self-reported personality and perceived personality from others (e.g., Funder, 1995; Borkenau et al., 2009; Chan et al., 2010; Todorov et al., 2015; Alaei and Rule, 2016). However, with regard to attributes without objective criteria, such as self-reported stress (Little et al., 2011), researchers employ self-other agreement or distinctive self-other agreement (Human et al., 2013) to measure facial judgment accuracy. According to the above definitions of accuracy, in the current research, the accuracy of facial attractiveness was calculated by self-other agreement.

According to the trait visibility effect (Funder and Dobroth, 1987; Watson et al., 2000) and the acquaintanceship effect (Funder, 1995), we hypothesized that people tend to give more accurate ratings of the facial attractiveness of female faces than of male faces. These gender differences arise because across many cultures, a woman’s attractiveness is important (Li et al., 2002; Shackelford et al., 2005), whereas a man’s status and resources are more crucial than his attractiveness (Buss and Schmitt, 1993; Sprecher et al., 1994). Therefore, in Studies 1 and 2, we explored the gender differences in judgment accuracy and metaperception accuracy on facial attractiveness. Study 3 was designed to investigate the cognitive mechanism of these gender biases. Participants were asked to give their ratings on the perceived wealth of the person depicted in each photo in Study 3. The accuracy of perceived economic status in Study 3 was calculated by the correspondence between the participants’ subjective perception of faces and the actual wealth ranking group.

## Study 1

This experiment was designed to explore the influence of the perceiver’s gender on accuracy in judging facial attractiveness. Considering that facial attractiveness carries additional significance for women (Luxen and Van De Vijver, 2006), people may be more accustomed to evaluating women’s attractiveness in everyday life, which motivates women to pay more attention than men to their attractiveness. Thus, we hypothesized that (1) people tend to give more accurate ratings of facial attractiveness for female faces than for male faces and (2) women tend to assess people’s facial attractiveness more accurately than men.

### Methods

#### Participants

A total of 90 students participated in Study 1 for payment, including 41 males (M_age_ = 24.32, SD_age_ = 3.66) and 49 females (M_age_ = 24.86, SD_age_ = 4.68). This study was approved by the internal review board of the Department of Psychology, Renmin University of China. Each participant signed an informed consent form and received monetary compensation for his or her time.

#### Stimuli

Another 119 undergraduate students (58 male and 61 female, age range 18–25 years) were recruited to have frontal shoulder-up pictures taken with a digital camera in front of a white background for use as stimuli. Before the photos were taken, they removed all accessories except glasses (if they could not finish the task without them). We asked the students to maintain a natural (neutral emotion) expression. After the photos were taken, they were asked to rate their attractiveness in the eyes of others of the same gender and different genders using a 9-point scale ranging from 1 (not attractive at all) to 9 (extremely attractive). We did not find a difference between their self-ratings of attractiveness in the eyes of others of the same gender and others of a different gender *t*(236) = −0.42, *p* = 0.678. All photographed participants consented to the use of their photos for our research purposes, including showing their pictures to other participants. All the faces were adjusted to the same size, 295 × 295 pixels.

#### Apparatus and Procedure

**FIGURE 1:**
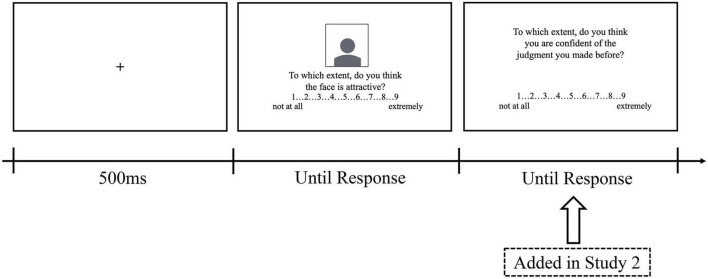
The order of events in a typical trial of Studies 1 and 2.

The experiment was conducted on a computer with E-prime 2.0. Participants were told to use their gut feeling to rate the attractiveness of each face photo. In a typical trial of the study, a fixation point was presented for 500 ms, and then a face photo was shown with a 9-point rating scale below it. Participants were asked to give their rating on the attractiveness of each face photo from 1 (not attractive at all) to 9 (extremely attractive). The experiment contained two blocks with a total of 238 trials, and each face photo was presented once in a block. The order of the photos was random. Participants started with a practice block of 8 trials to familiarize them with the task. Between the two blocks, the participants were allowed to take a break and started the next block on their own if they thought they were ready for it (see Figure 1). Considering that one participant rated the same target twice during the study, we used the mean rating for each face as the other-rating of the face.

In the review of Devos et al. (2013), self-other agreement is a relative phenomenon that refers to a degree of discrepancy between self-ratings and other-ratings. In previous research, self-other agreement was operationalized as the absolute difference of self and other ratings (Atwater and Yammarino, 1992, 1997; Bernieri et al., 1994; Lee and Carpenter, 2018; Kim et al., 2019) in addition to correlation (Borkenau and Liebler, 1993; Rogers et al., 2018). In the present research, we standardized the ratings of attractiveness for each face by subtracting other-ratings from self-ratings. Specifically, when a participant rated the face of someone of the same gender, the other-rating of attractiveness for this face was subtracted from the self-rating in the eyes of others with the same gender and vice versa. Thus, the standardized rating scores, which refer to rating accuracy, ranged from −8 to 8, with higher scores indicating that participants rated the target’s attractiveness lower than the target’s self-ratings. The absolute value indicates the difference between self-rating scores and other-rating scores. To be more specific, a higher absolute value indicates that participants rated the target’s attractiveness lower than the target’s self-ratings. Positive or negative values suggest whether participants underestimated or overestimated facial attractiveness compared to self-ratings. All subsequent analyses were based on the standardized data.

### Results

**FIGURE 2:**
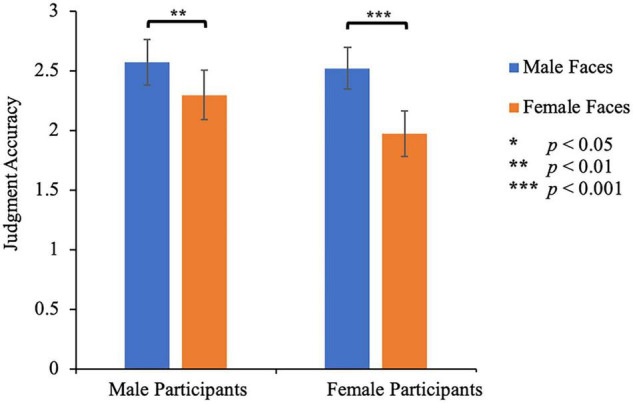
Rating accuracy of facial attractiveness in Study 1. Standardized rating scores as a subtraction of self-rating and other-rating facial attractiveness in Study 1. Error bars represent 1 S.E. of the means.

Mean standardized ratings were submitted to a 2 (participant’s gender: male, female) × 2 (facial gender: male, female) mixed-design measures ANOVA with face gender as a within-subject factor (Figure 2). The main effect of face gender was significant, *F*(1, 88) = 62.07, *p* < 0.001, η_*p*_^2^ = 0.414, indicating that female faces (2.14 ± 0.14) were judged more accurately than male faces (2.55 ± 0.13). The main effect of participant’s gender was not significant, *F*(1, 88) = 0.50, *p* = 0.483, η_*p*_^2^ = 0.006, indicating that the influence of the participant’s gender on judgment accuracy was relatively limited. More importantly, there was a significant interaction between the participant’s gender and facial gender, *F*(1, 88) = 6.87, *p* = 0.010, η_*p*_^2^ = 0.072. Male participants rated female faces (2.30 ± 0.21) more accurately than male faces (2.57 ± 0.19), *p* = 0.001. Female participants also rated female faces (1.97 ± 0.19) more accurately than male faces (2.52 ± 0.17), *p* < 0.001. These results indicated that compared to the self-ratings of the targets, the participants’ ratings tended to underestimate the targets’ attractiveness. More importantly, all participants showed higher rating accuracy in judging the attractiveness of female faces. In addition, for male faces, male participants and female participants had similar rating accuracy (2.57 ± 0.19 vs 2.30 ± 0.021, *p* = 0.845), while for female faces, male participants and female participants also had similar rating accuracy (2.52 ± 0.17 vs 1.97 ± 0.19, *p* = 0.255).

## Study 2

Study 1 found that participants tend to rate female faces’ attractiveness more accurately than male faces, which confirms hypothesis 1. Thus, Study 2 was designed to retest these findings. Moreover, to explore whether participants were aware of their rating accuracy, we added a confidence-rating task to the experiment and calculated the correlation between confidence rating and rating accuracy.

### Methods

#### Participants

A total of 50 students at Nankai University participated in Study 2, including 25 males (M_age_ = 20.88, SD_age_ = 1.92) and 25 females (M_age_ = 20.68, SD_age_ = 1.22). Each participant signed an informed consent form and received monetary compensation for his or her time.

#### Stimuli, Apparatus, and Procedure

The stimuli and procedure were identical to those in Study 1, with one exception. Participants were asked to rate their confidence after giving their attractiveness rating in each trial to explore whether raters were aware of the accuracy of their judgments. Specifically, after rating the attractiveness of the target face photo, a 9-point scale was shown with the question, “To what extent do you think you are confident of the judgment you made before?” Participants could respond from 1 (not confident at all) to 9 (extremely confident).

### Results

#### Attractiveness Rating

**FIGURE 3:**
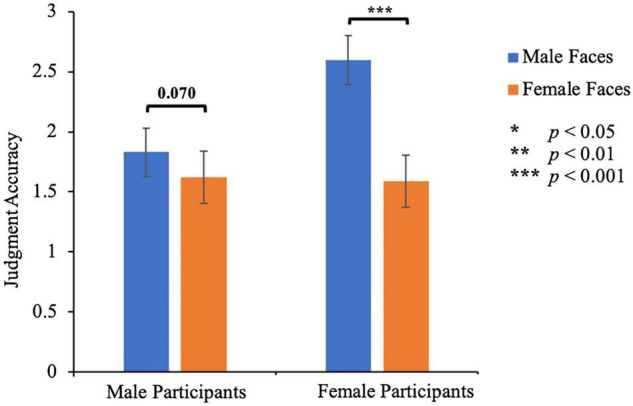
Rating accuracy of facial attractiveness in Study 2. Error bars represent 1 S.E. of the means.

Mean standardized ratings were submitted to a 2 (participant’s gender: male, female) × 2 (facial gender: male, female) mixed-design measures ANOVA with facial gender as a within-subject factor (Figure 3). The main effect of facial gender was significant, *F*(1, 48) = 57.30, *p* < 0.001, η_*p*_^2^ = 0.544, indicating that female faces (1.61 ± 0.15) were judged more accurately than male faces (2.21 ± 0.14). The main effect of participant’s gender was not significant, *F*(1, 48) = 1.67, *p* = 0.203, η_*p*_^2^ = 0.034. There was a significant interaction between the participant’s gender and facial gender, *F*(1, 48) = 24.50, *p* < 0.001, η_*p*_^2^ = 0.338. Specifically, male participants rated female faces (1.62 ± 0.22) almost as accurately as male faces (1.83 ± 0.20), *p* = 0.070. Female participants rated female faces (1.59 ± 0.22) more accurately than male faces (2.60 ± 0.20), *p* < 0.001. These results confirmed that compared to the self-ratings of the targets, the participants’ ratings tended to underestimate the facial attractiveness of male and female target faces. Similar to Study 1, all participants showed higher accuracy in judging the attractiveness of female faces. In addition, for male faces, male participants gave more accurate ratings (1.83 ± 0.20) than female participants (2.60 ± 0.20, *p* = 0.010), while for female faces, male participants and female participants had similar rating accuracy (1.62 ± 0.22 vs 1.60 ± 0.22, *p* = 0.307).

#### Confidence Rating

We conducted a 2 (participant’s gender) × 2 (facial gender) mixed-design measures ANOVA with facial gender as a within-subject factor. The main effect of facial gender was not significant, *F*(1, 48) = 2.41, *p* = 0.191, η_*p*_^2^ = 0.048. The main effect of participant’s gender was also not significant, *F*(1, 48) = 1.10, *p* = 0.307, η_*p*_^2^ = 0.022. The interaction between the participant’s gender and facial gender was not significant, *F*(1, 48) = 3.79, *p* = 0.058, η_*p*_^2^ = 0.073. These results suggested that male and female participants were not aware of their own accuracy in judging the attractiveness of others based on the facial appearance of different genders. Moreover, there was a significant but weak positive correlation between rating confidence and judgment accuracy (r = 0.27, *p* = 0.006). Specifically, the correlation between confidence ratings and rating accuracy was significant when female participants rated male faces (*r* = 0.52, *p* = 0.008). In other words, when male participants rated male (*r* = 0.23, *p* = 0.275) and female faces (*r* = 0.21, *p* = 0.323) or female participants rated female faces (*r* = 0.28, *p* = 0.173), they lacked a clear awareness of their rating accuracy.

## Study 3

Studies 1 and 2 found that participants rated the attractiveness of female faces more accurately than that of male faces. There are two possible explanations: (1) the self-ratings of males are higher than those of females, or (2) the more people care about females’ facial attractiveness, the more accurately they are able to rate this trait. Thus, we compared the self-ratings of male and female faces and found that there was no significant difference between males’ and females’ self-ratings in the eyes of others of the same gender [*t*(117) = 0.23, *p* = 0.985] or a different gender [*t*(117) = −0.74, *p* = 0.246]. We conducted Study 3 to test the second explanation. Elder (1969) and Udry (1977) found that physical attractiveness has a “market value” for females, while males tend to be evaluated in terms of their status (Wade, 1988). This indicates that people pay more attention to the attractiveness of females, while they care more about the social status or wealth of males. Therefore, this experiment was designed to test whether participants could rate the wealth of males more accurately than that of females based on facial appearance.

### Methods

#### Participants

Another 50 students at Nankai University participated in Study 3, including 25 males (M_age_ = 21.48, SD_age_ = 2.63) and 25 females (M_age_ = 20.12, SD_age_ = 1.90). Each participant signed an informed consent form and received monetary compensation for his or her time.

#### Stimuli

Using the Chinese Rich List (China Fuhao List, 2019), we selected 72 photos of faces (36 male and 36 female) as the targets and excluded all famous people, such as Jack Ma. Considering that the age of faces might affect the wealth ratings, we balanced the numbers of male and female faces in each age group and selected faces across a variety of age groups (see Table 1).

**TABLE 1:** Number of each age group of targets in Study 3.

Age group	Number of female/male faces
30–39	9/9
40–49	9/9
50–59	10/10
60 above	8/8

All the facial photos of wealthy people were found online. In the photos, their eyes look straight ahead. All the photos were manipulated to show the person from the shoulder up, against a white background and of the same size (295 × 295 pixels). Unlike in Studies 1 and 2, we used an objective standard of wealth rather than subjective self-reported attractiveness to compare with the participants’ ratings when examining rating accuracy. In Study 3, based on wealth, we sorted wealthy people from high to low and divided them into nine equal groups (1-most wealthy group, 9-least wealthy group). By using the chi-square test, we ensured that in each wealth group, neither age, χ^2^(264) = 278.40, *p* = 0.260, nor gender made a difference, χ^2^(8) = 13.00, *p* = 0.112. We conducted a gender (facial gender: male, female) × group ANOVA and found that the main effect of facial gender was not significant, *F*(1, 54) = 0.02, *p* = 0.878, η_*p*_^2^ = 0.000. The main effect of group was significant, *F*(8, 54) = 41.60, *p* < 0.001, η_*p*_^2^ = 0.860. The interaction of facial gender and group was also not significant, *F*(8, 54) = 0.07, *p* > 0.999, η_*p*_^2^ = 1.000. These results suggested that the gender differences in the fortune of each group are negligible (see Table 2).

**TABLE 2:** Average fortune of each group of targets in Study 3.

Group	Average fortune (billion RMB)	Average fortune of females (billion RMB)	Average fortune of males (billion RMB)
1	143.32	147.38	141.96
2	95.22	89.55	97.11
3	59.22	60.56	58.41
4	42.35	40.34	43.56
5	28.91	28.50	29.57
6	18.25	18.96	17.83
7	13.26	13.29	13.00
8	8.95	8.93	9.00
9	4.20	2.80	6.53

#### Procedure and Design

Guessing people’s wealth based on facial appearance is impossible and lacks objective standards; thus, the participants saw an overview picture containing all the faces arranged randomly before the rating began. All the faces were arranged randomly into six lines, with 12 pictures per line. We made 10 versions of the overview pictures, one of which was shown randomly in one experiment. The picture was presented for 8,000 ms so that participants could establish an overall impression of these faces. The duration of the display was determined by a pilot study in which we asked people to look at the photos and react when they thought they were ready for the next step. Then, they were told, “Each face belongs to 1 of the 9 richness wealth groups; please use your gut feeling to guess which group each face photo is in”. Participants started with a practice block with eight trials to familiarize them with the task. The eight pictures used in the practice block (four male and four female) were chosen from the Chinese Rich List, which was the same source as the 72 experimental figures. However, they were not included in the 72 experimental figures, and the ratings of the eight faces were not included in the following analysis.

In a typical trial of the study, a fixation point was presented for 500 ms, and then a face photo was shown with a 9-point rating scale below it. Participants were asked to give their ratings on the wealth of the person depicted in each photo from 1 (most wealthy group) to 9 (least wealthy group). They were then asked to rate their confidence in the judgment they had made. Finally, they were asked to answer the question, “Are you familiar with this person?” by pressing 1 (no) or 9 (yes). The experiment contained two blocks with a total of 152 trials, and each face photo was presented once in a block. The order of the presentation was random. Between the two blocks, the participants were allowed to take a break and start the next block on their own if they thought they were ready for it. Considering that one participant rated the same target twice during the study, we used the mean rating for each face as the other-rating of the face. In the present research, we standardized the ratings of wealth for each face by subtracting other-ratings from objective ratings. Thus, standardized rating scores ranged from −8 to 8, with higher scores indicating that participants rated the target’s wealth lower than the actual rating. The absolute value indicates the difference between objective rating scores and other-rating scores. To be more specific, a higher absolute value indicates that participants rated the target’s wealth ranking lower than the target’s actual wealth ranking. Positive or negative values suggest whether participants underestimated or overestimated the wealth rankings compared to an objective standard. All subsequent analyses were based on the standardized data.

To avoid the influence of familiarity, we excluded the data if the participant recognized the face in both blocks. Thus, some trials (2.71%) were not included in the following analysis.

### Results

#### Richness Rating

**FIGURE 4:**
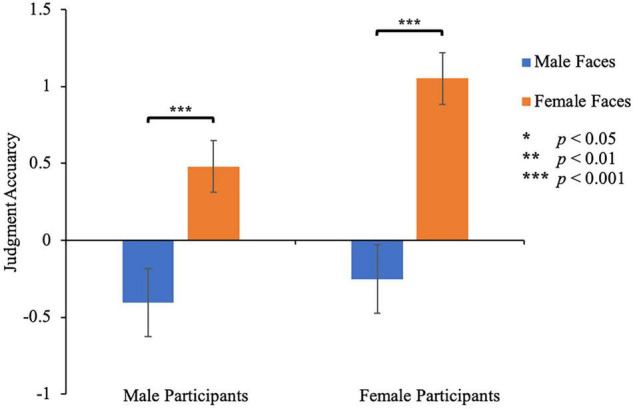
Rating accuracy of wealth in Study 3. Error bars represent 1 S.E. of the means.

Mean standardized ratings were submitted to a 2 (participant’s gender) × 2 (facial gender) mixed-design measures ANOVA with facial gender as a within-subject factor (Figure 4). The main effect of facial gender was significant, *F*(1, 48) = 84.17, *p* < 0.001, η_*p*_^2^ = 0.631, indicating that people give more accurate ratings of wealth for male faces (−0.33 ± 1.10) than for female faces (0.77 ± 0.88). The main effect of the participant’s gender was not significant, *F*(1, 48) = 2.08, *p* = 0.156, η_*p*_^2^ = 0.042. There was no significant interaction between the participant’s gender and facial gender, *F*(1, 48) = 3.08, *p* = 0.086, η_*p*_^2^ = 0.060. Specifically, male participants rated male faces (−0.41 ± 0.22) more accurately than female faces (0.48 ± 0.17), *p* < 0.001. Female participants also rated male faces (−0.25 ± 0.22) more accurately than female faces (1.05 ± 0.17), *p* < 0.001. These results indicated that all participants showed higher rating accuracy in judging the wealth of male faces than that of female faces. Additionally, for male faces, male raters and female raters had similar accuracy (−0.41 ± 0.22 vs −0.25 ± 0.22, *p* = 0.626), while for female faces, male raters gave more accurate ratings (0.48 ± 0.17) than female raters (1.05 ± 0.17, *p* = 0.020).

#### Confidence Rating

We conducted a 2 (participant’s gender) × 2 (facial gender) mixed-design measures ANOVA with facial gender as a within-subject factor. The main effect of facial gender was not significant, *F*(1, 48) = 0.02, *p* = 0.902, η_*p*_^2^ = 0.000. The main effect of participant’s gender was not significant, *F*(1, 48) = 0.99, *p* = 0.325, η_*p*_^2^ = 0.020. The interaction of the participant’s gender and facial gender was also not significant, *F*(1, 48) = 0.70, *p* = 0.407, η_*p*_^2^ = 0.014. These results suggested that male and female participants were not aware of their own accuracy in judging others’ wealth based on the facial appearance of different genders. Moreover, the correlation between confidence ratings and rating accuracy was not significant, *r* = 0.01, *p* = 0.962. Specifically, whether male participants rated male (*r* = −0.05, *p* = 0.831) and female faces (*r* = 0.28, *p* = 0.174) or female participants rated male (*r* = 0.09, *p* = 0.662) and female faces (*r* = −0.13, *p* = 0.532), they had low awareness of their rating accuracy.

## General Discussion

The present study showed that people give more accurate judgments of the facial attractiveness of female faces than of male faces and give more accurate wealth judgments for male faces than for female faces. To our knowledge, the current research is the first to show gender biases in the accuracy of impressions formed from faces. This indicates an important role of facial gender in shaping accurate first impressions.

The differences in judgment accuracy of male and female faces may be caused by differences in traditional gender roles. From an evolutionary perspective, these gender biases have been linked to the production and survival of offspring. A man’s reproductive potential is related more to his (economic) resources. In contrast, a woman’s reproductive potential is associated more closely with her health, which may be related to physical attractiveness (Luxen and Van De Vijver, 2006). Thus, females might be more familiar with others’ evaluations of their own facial attractiveness and thus achieve a higher level of consistency on self-other agreement. These results are also consistent with previous findings that facial gender is a salient facial cue in face processing and has an effect on other types of information (e.g., expression) processing (Liu et al., 2017). Moreover, Maner et al. (2003) found that both male and female observers selectively focus on physically attractive female targets according to the targets’ facial photos, suggesting that people care more about female facial attractiveness than male facial attractiveness. The more attention that is paid to female facial attractiveness, the more accurate the judgments that can be made based on facial appearance.

In contrast to the findings about female faces in Studies 1 and 2, Study 3 revealed that people tend to rate perceived socioeconomic status (SES) more accurately for male faces than for female faces. In mate selection, SES is of great significance to males since females are more attentive to resources that can be invested in themselves and their offspring (Wang et al., 2018). Thus, on the one hand, males will expend more effort to increase their SES and recognize SES differences between themselves and competitors so that they can attract potential mates. On the other hand, females will seek as much evidence as possible to confirm their judgment of males’ SES to help them “make a good choice”. Moreover, because the number of male billionaires is larger than that of females all over the world (Wai, 2014; Forbes, 2022) and there is more media news or information related to wealthy males than to wealthy females, people may learn more useful cues to help them rate males’ SES, even using only faces. Therefore, people’s gender stereotypes are enhanced when SES is highly correlated with males in society. Similar gender bias is also found in research on how masculine facial cues play a key role in competence impressions (Oh et al., 2019). When people evaluate traits or personalities, the more evidence they accumulate and the more information they have observed and mastered, the higher the accuracy of their judgments and evaluations will be (Watson et al., 2000; Biesanz et al., 2007). These findings provide cross-validation of our hypothesis that people may pay more attention to the characteristics that are consistent with gender roles (e.g., the attractiveness of women, the socioeconomic status of men), thus accumulating more evidence that helps them make more accurate judgments.

The current findings regarding gender bias show the great social influences on gender differences. The higher accuracy of judgments of the facial attractiveness of female faces and of the wealth of male faces indicates that people can make relatively accurate judgments about these factors based only on faces. More importantly, it suggests that when the characteristics are consistent with gender stereotypes and are emphasized by society, people assign more attention to the characteristics of the gender. As a result, by accumulating more experience and evidence, people can make more accurate judgments. On the positive side, people can quickly establish a relatively accurate impression of some characteristics that fit gender stereotypes to benefit their daily life interactions. However, the restricted accuracy of impressions based on face photos should receive more research attention. On the negative side, people put little effort into learning about characteristics that conflict with gender stereotypes, which might aggravate gender stereotypes across society. In addition, in Studies 2 and 3, we found that males rated characteristics that conflict with gender stereotypes more accurately than females did, which suggests that males might be affected less by gender stereotypes. This finding could be further examined in future research.

The analysis of confidence ratings implies that although the participants were able to make relatively accurate judgments, they may have struggled to be aware of their judgment accuracy. Participants might not realize whether they have extracted useful information from faces to help them make judgments. In addition, it is possible that they might not be sure of the gap between their own standards and external standards while giving their ratings. However, in Study 2, the significant moderate correlation between confidence ratings and rating accuracy when female participants rated male faces is interesting and is in line with research showing that females exhibit higher levels of interpersonal sensitivity than males (Chan et al., 2010). Despite female participants’ higher accuracy when rating female faces, they had a clearer awareness when rating male faces. When rating male faces, even though male participants rated them more accurately, they failed to recognize their rating accuracy. However, we did not find a similar result in Study 3. Overall, these results show that although gender bias exists in terms of judgment accuracy, people do not have a relatively clear awareness of their rating behaviors and the gender bias of their judgments. This means that during the rating process, people might have underlying evaluation references that they are unaware of, which could be explored more thoroughly in the future.

## Conclusion

The present study demonstrates that people evaluate females’ attractiveness and males’ perceived SES more accurately when looking at faces. Thus, we conclude that people evaluate the traits that they pay attention to more accurately based on facial appearance. In sum, these results reveal the effect of gender stereotypes on the judgment accuracy of impressions from faces. Accurate first impressions have a long-term effect on social relationship development (Human et al., 2013). The causes of this effect require more research. On the one hand, the present study illustrates that just by looking at faces, people can form relatively accurate impressions about traits that fit gender stereotypes. On the other hand, it shows the long-term and intensive impacts of social attitudes such as gender stereotypes on our daily life and social interactions.

## Data Availability Statement

The data that support the findings of this study are openly available at https://osf.io/dzfvp.

## Ethics Statement

The studies involving human participants were reviewed and approved by the internal review board of the Department of Psychology, Renmin University of China. The patients/participants provided their written informed consent to participate in this study.

## Author Contributions

YQ designed and performed the experiments and wrote the manuscript. JY performed the experiments, analyzed the data, and wrote the manuscript. Both authors contributed to the article and approved the submitted version.

## Conflict of Interest

The authors declare that the research was conducted in the absence of any commercial or financial relationships that could be construed as a potential conflict of interest.

## Publisher’s Note

All claims expressed in this article are solely those of the authors and do not necessarily represent those of their affiliated organizations, or those of the publisher, the editors and the reviewers. Any product that may be evaluated in this article, or claim that may be made by its manufacturer, is not guaranteed or endorsed by the publisher.
